# Prognostic role of overexpressed Bromodomain and extra-terminal family in ovarian cancer

**DOI:** 10.7150/jca.69574

**Published:** 2022-03-14

**Authors:** Mandika Chetry, Adheesh Bhandari, Yue Lin

**Affiliations:** 1 Department of Reproductive Medicine Center, The First Affiliated Hospital of Wenzhou Medical University, Wenzhou, Zhejiang Province, 325000, China; 2 Department of Oncology, The First Affiliated Hospital of Shantou University Medical College, Shantou, Guangdong, China; 3 Department of Breast Surgery, The First Affiliated Hospital of Wenzhou Medical University, Wenzhou, Zhejiang Province, China; 4 Department of General Surgery, Breast and Thyroid Unit, Primera Hospital, Kathmandu, Nepal

**Keywords:** Bromodomain and extra-terminal, BET, prognosis, ovarian cancer

## Abstract

**Background:** BET family proteins have a role as epigenetic readers to accelerate the transcription of target genes. Several studies have shown that the BET protein family played important roles in several biological processes. Although, the prognostic influence of individual BET genes family in ovarian cancer patients remains unclear.

**Methods:** We investigated BET mRNA prognostic roles subtypes in ovarian cancer patients by means of the KM plotter database. The BET mRNA expression and protein in cancer and normal ovarian cells was determined using qRTPCR and western blot. We used the HPA database to look at the protein expression profiles in normal and cancer tissues for this study.

**Results:** Among BET members, mRNA expression BRD2 showed improve OS in all the ovarian malignancy patients, serous patients, stage III and IV, grade II and grade III, TP53 mutated ovarian cancer patients, as well as all patients treated with Platin based chemotherapy. As for BRD3, we found that BRD3 expression was related to better OS in endometrioid ovarian carcinoma and stage III+IV ovarian carcinoma patients, as well as all patients managed with Taxol and concurrent Taxol+Platin based chemotherapy. In addition, BRDT was associated with better OS in all ovarian carcinoma patients, grade I and grade III, all clinical stage (I+II, III+IV) patients, as well as all patients cured with Taxol and concurrent Taxol+Platin chemotherapy.

**Conclusion:** We conclude that high expression of BRD2, BRD3, and BRDT predicted a better prognosis. mRNA expression of BET family is considerably associated with the prognosis of ovarian carcinoma and individual BET family gene could act as a predictive prognostic indicator in ovarian carcinoma.

## Introduction

**Figure 1:**
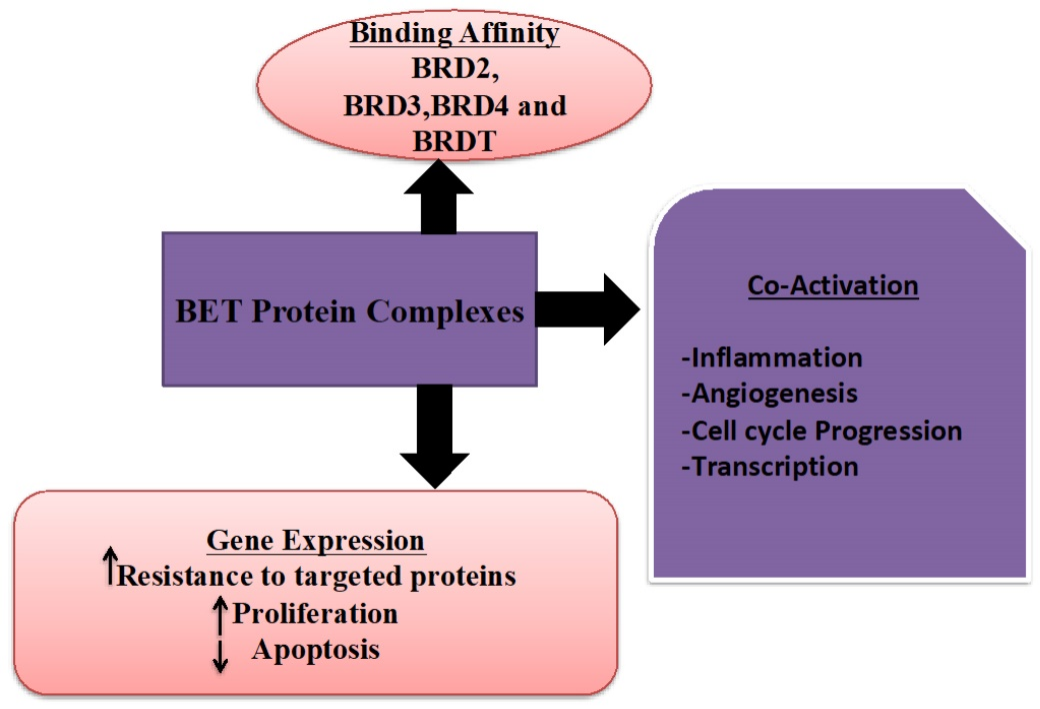
The role and types of BET protein family.

Ovarian carcinoma is the fifth important source of gynecological cancers to death in women in the United States 1. It is a diverse and genomically complex disease. The standard cure for the advanced stage of ovarian malignancy is a pattern of surgery and chemotherapy 2. Despite early detection and breakthroughs in chemotherapy, the 5-year survival rate for women with advanced ovarian malignancy persists at only 30% 3. A more frequently abundant number of cases are diagnosed after cancer has metastasized. In addition, screening trials have shown non-relevant outcomes on mortality and have displayed adverse effects; positive findings revealed asymptomatic females often found with benign pelvic conditions or normal ovarian condition on the surgical investigation, and cancers are often missed with screening 4, 5. Therefore, it is imperative to identify the predictive biomarkers for ovarian cancer prognosis and develop novel therapeutic options to improve these patients' clinical results. Bromodomain and extra-terminal (BET) family proteins (Figure 1), which have a role as epigenetic readers to accelerate the transcription of target genes by recognition of acetylated histone tails and activate the RNA polymerase II 6. The BET protein contains of four members, including BRD2, BRD3, BRD4, and BRDT 7. Several studies have revealed that the BET protein family played vital functions in regulating cell proliferation, invasion, apoptosis, and metastasis potentials 8-10. However, little is known about the distinctive impact on prognosis in ovarian cancer patients of BET family gene expression. In the present analysis, we comprehensively retrieved the prognostic roles of distinct mRNA expression of BET gene in ovarian carcinoma patients by means of the Kaplan-Meier plotter database. Using the Kaplan-Meier plotter, this work attempts to establish the predictive value of specific mRNA expression of BET members in ovarian tumor patients according to distinct clinicopathological parameters (including pathological grade, clinical stages, and TP53 mutation status) and chemotherapy (KM plotter). Furthermore, we discovered the difference in BET expression by comparing ovarian cancer cell lines to normal ovarian cell lines, it was possible to find a more useful prognostic biomarker for predicting the prognosis of individuals with ovarian malignancies. In HPA, cell lines are explained using a programmed image examination based on immunohistochemistry (IHC) labeling. A comparable scoring approach has also been used to describe the level of expression. As a result, the database strongly encourages the use of protein expression to define potential biomarker patterns that indicate if a specific protein could be utilized as a biomarker.

As a result, we conducted a thorough investigation on the predictive impact of BET family members in ovarian cancer patients, in addition to their relationships with clinical stages, pathological grades, and TP53 subtypes along with applied chemotherapy. In addition, to find a further useful prognostic biomarker for predicting the prognosis of ovarian cancer patients, there was a difference in BET member expression amongst ovarian cancer cell lines and normal ovarian cell lines, as well as a difference in expression between ovarian cancer tissues and normal ovarian tissues.

## Methods and Materials

To explore the online database, we used the Kaplan-Meier plotter (http://kmplot.com/analysis/) 11 the relationship between distinctive BET family mRNA level and overall survival (OS) of ovarian carcinoma patients. Presently, this databank is capable of assessing the outcome of 54,675 genes of survival in breast cancer 11, ovarian cancer 12, lung cancer 13, as well as gastric carcinoma. In this database, 2,190 ovarian carcinoma patients gene expression data and OS information were taken from Gene Expression Omnibus, Cancer Biomedical Informatics Grid, and The Cancer Genome Atlas cancer datasets 13. Furthermore, the database included clinicopathological data, such as histology, grade, stage, TP53 mutation status, and applied chemotherapy for ovarian cancer patients. In short, four BET sub-members (BRD2, BRD3, BRD4, and BRDT) were recorded into the database (http://kmplot.com/analysis/index.php? p = service & cancer = ovarian) to get Kaplan-Meier survival plots. The specific BET genes expression cut-off values were obtained according to gene mRNA expression using the auto select best cut-off value amongst the particular ovarian cancer models. Then BET expressions were categorized into “low” and “high” according to the relationships among the expression values with recognized cutoffs. Hazard ratio (HR) and 95% confidence intervals (95% CI), as well as log-rank P, was analyzed and shown on the web page. *P*-value < 0.05 was considered to be statistically interrelated.

The primary case inclusion criteria were as follows: (1) non- pregnant; (2) patients with serous ovarian cancer with no other organs severely affected; (3) patients who had undergone hysterectomy and bilateral salpingo-oophorectomyhad not received radiotherapy; (3) patients with no medical history of other malignant tumor; (4) patients with complete clinical baseline characteristics; and (5) non- breast-feeding women. The primary case exclusion criteria were as follows: (1) patients with other malignancies or a history of other malignancies; (2) patients suffering from serious illness such as heart failure, stroke or chronic renal failure.

### The Human Protein Atlas

Plots of human tissue proteome using quantitative transcriptomics at the tissue and organ level, as well as protein profiling using microarray-based immunohistochemistry, presenting evidence on cell-specific localization in 44 diverse normal tissues and organs, together with 20 different kinds of cancers with the help of the Human Protein Atlas (http://www.proteinatlas.org/) 14. As for the immunohistochemistry, all used antibodies undergo the standard antibody validation, including the human protein atlas antibodies (HPA-antibodies), commercially available antibodies (CAB-antibodies), and so on. We to investigate the BET family genes protein expression in ovarian cancer tissues and normal ovary tissues utilize this database.

### Cell Lines and Cell Culture

ES2, OVCAR-3, and A2780 human ovarian cancer cell lines and IOSE80 normal ovarian epithelial cell line were obtained from (Fuheng, Shanghai, China) were cultivated in Dulbecco's modified Eagle's medium (DMEM) (Biosharp, China) augmented with 10% fetal bovine serum (Biosharp, China) and 100 U/mL penicillin and 100 g/mL str (Gibco, Thermo Fisher Scientific).

### Western blot analysis

To acquire entire cell lysates, the cells were mixed with lysis buffer (Beyotime Biotechnology, Shanghai, China). A bicinchoninic acid protein examine kit was used to determine the protein content (Beyotime Biotechnology, Shanghai, China). 12 % sodium dodecyl sulphate polyacrylamide gel electrophoresis was used to detached equal amounts of protein on each lane. Following that, the proteins were transported to a polyvinylidene fluoride membrane (Millipore, Boston, MA, USA). BRD2, BRD3, BRD4, and BRDT (1:1000, Abcam, San Francisco, CA, USA), were incubated overnight on the membrane. The membrane was rinsed in TBST buffer. The second antibody was added to the membrane and detected with Logene-i YG2006 image acquisition system.

### RNA extraction and quantitative real‐time PCR (qRT‐PCR)

RNA extraction and quantitative real-time PCR (qRTPCR) total RNA was extracted by using the TRIzol reagent (Biosharp, China) agreeing to the company's guidelines, and cDNAs were generated using the reverse transcription. For qRTPCR, the following conditions were used: With the PCR Kit, 94.0°C for 30 seconds was followed by 39 cycles of 94.0°C for 5 seconds and 60.0°C for 30 seconds (Biosharp, China). The internal control for GAPDH was set for gene quantification. With qRT-PCR analysis, the number of technical and biological duplicates for each gene was at least three times. The primers that were used in this analysis were as follows:

Human BRD2: Forward: 5'-GGAAACATCAGTTCGCATGGC-3'; Reverse: 5'-CACTCTGAAGCAGCCCAATAA-3'.

HUMAN BRD3: Forward: 5'-TGCAAGCGAATGTATGCAGGA-3'; Reverse: 5'-CATCTGGGCCACTTTTTGTAGAA-3'.

HUMAN BRD4: Forward: 5'-GAGCTACCCACAGAAGAAACC-3'; Reverse: 5'-GAGTCGATGCTTGAGTTGTGTT-3'.

HUMAN BRDT: Forward: 5'-CTGTTGACGTTAATGCTTTGGG-3'; Reverse: 5'-CACAACTTCGTGATCTGGAGG-3'.

Human GAPDH: Forward: 5'-GGACCTGACCTGCCGTCTAG-3'; Reverse: 5'-GTAGCCCAGGATGCCCTTGA-3'.

### Statistical analysis

To determine significance, one-way ANOVA or two-tailed unpaired Student's t-tests were utilized. The data is presented as a mean standard deviation of the mean (SEM). *P* values less than 0.05 were used to determine statistical significance. For all statistical studies, Graph Pad Prism6.0 software was utilized.

## Results

**Figure 2:**
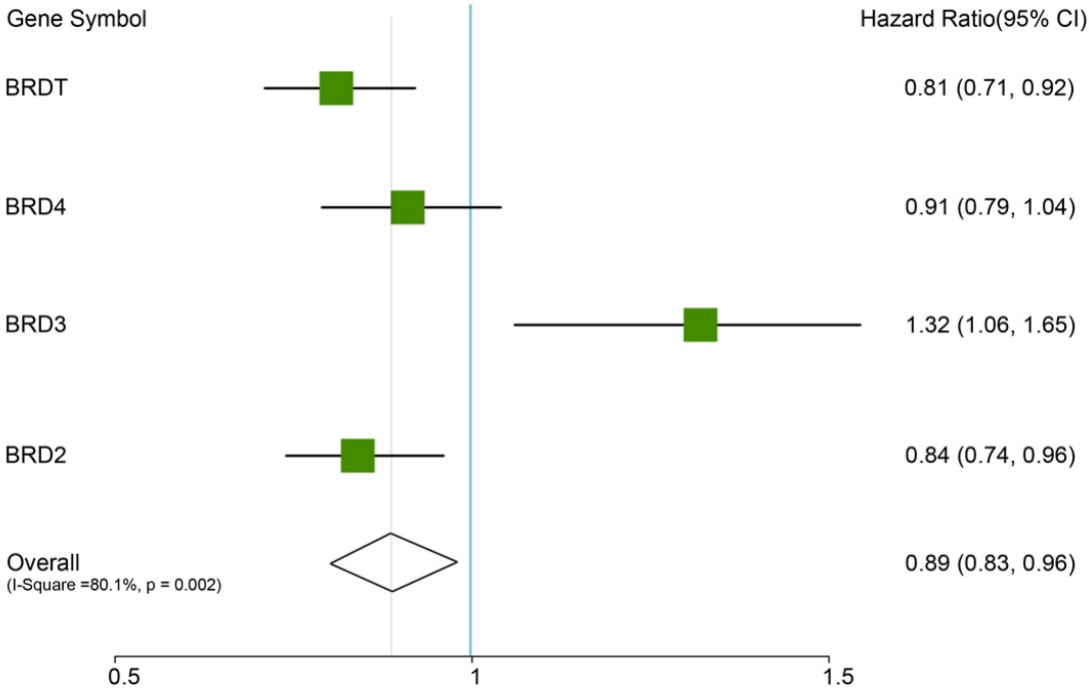
The prognostic HRs value of individual BRDs members in all ovarian cancer in www.kmplot.com.

**Figure 3:**
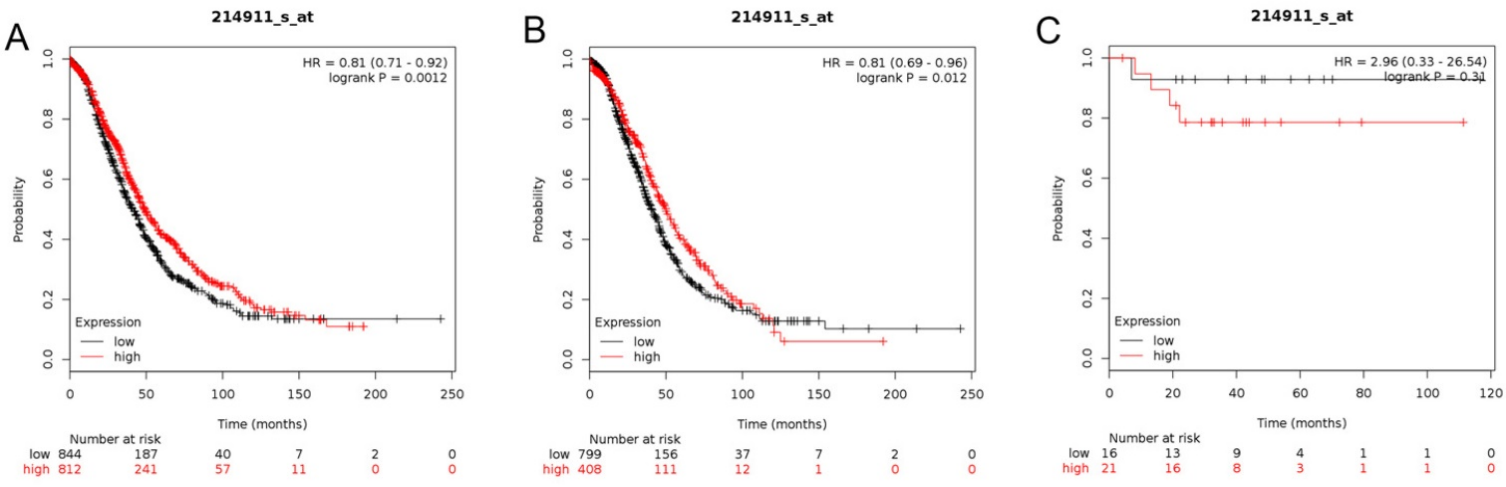
** The prognostic value of BRD2 expression in ovarian cancer.** The prognostic value of *BRD2* expression in www.kmplot.com. Affymetrix ID for BRD2: 214911_s_at. OS curves were plotted for (**A**) all the patients (*n*=1656), (**B**) serous cancer patients (*n*=1207), and (**C**) endometrioid cancer patients (*n*=37).

In this analysis, the prognostic values of the BETs members were obtained at www.kmplot.com (Figure 2). We firstly evaluated the prognostic value of BRD2 mRNA expression in the database (Figure 3). Affymetrix ID for BRD2: 214911_s_at. BRD2 mRNA expression exhibited significantly better association with OS among all the ovarian cancer patients together with serous histological subgroup, HR =0.81 (0.71 - 0.92), *P*=0.0012 (Figure 3A), HR =0.81 (0.69 - 0.96), *P*=0.012 (Figure 3B). Nevertheless, BRD2 mRNA high expression of endometrioid ovarian cancer did not display any association with OS, HR = 2.96 (0.33 - 26.54), *P*=0.31 (Figure 3C).

**Figure 4:**
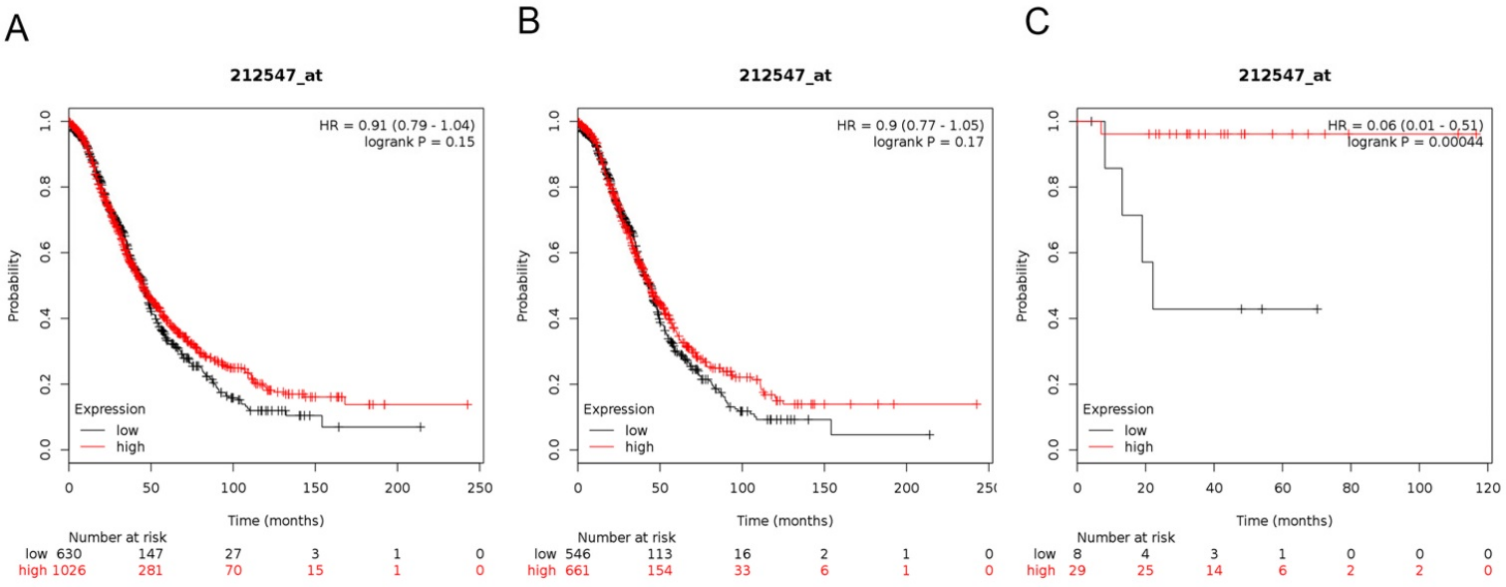
**The prognostic value of BRD3 expression in ovarian cancer.** The prognostic value of *BRD3* expression in www.kmplot.com. Affymetrix ID for *BRD3*: 212547_at. OS curves were plotted for (**A**) all the patients (*n*=1656), (**B**) serous cancer patients (*n*=1207), and (**C**) endometrioid cancer patients (*n*=37).

Next, we demonstrated the BRD3 prognostic value in the database (Figure 4). The Affymetrix ID for BRD3: 212547_at. mRNA expression of BRD3 exhibited no association with OS in all ovarian carcinoma as well as in serous ovarian carcinoma patients, HR= 0.91 (0.79 - 1.04), *P*= 0.15 (Figure 4A); HR= 0.9 (0.77 - 1.05), *P*= 0.17 (Figure 4B). However, high mRNA expression of BRD3 for endometrioid ovarian cancer was associated with favorable OS, HR= 0.06 (0.01 - 0.51), *P*= 0.00044 (Figure 4C).

**Figure 5:**
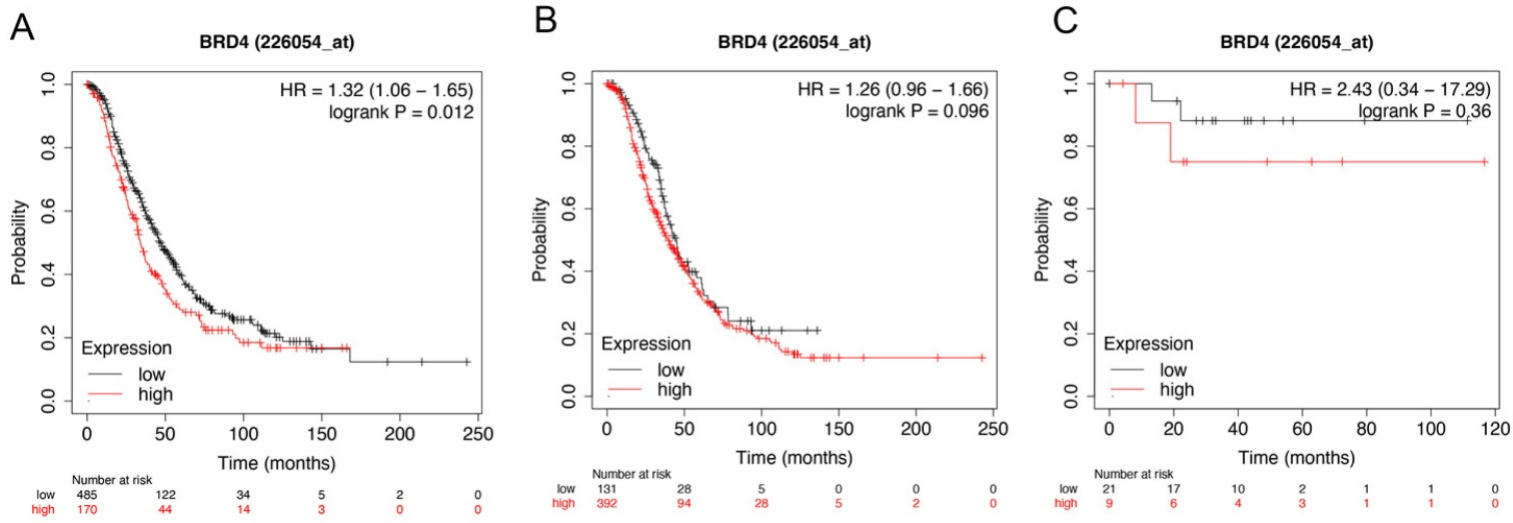
** The prognostic value of *BRD4* expression in ovarian cancer.** The prognostic value of *BRD4* expression in www.kmplot.com. Affymetrix ID for *BRD4*: 226054_at. OS curves were plotted for (**A**) all the patients (*n*=655), (**B**) serous cancer patients (*n*=523), and (**C**) endometrioid cancer patients (*n*=30).

Figure 5 showed the prognostic significance of BRD4 in the database. Affymetrix ID for BRD4: 226054_at. High BRD4 mRNA expression had worse OS for all ovarian cancer patients, HR =1.32 (1.06 - 1.65), *P*=0.012 (Figure 5A). However, expression of BRD4 mRNA in serous and endometrioid cancer patients exhibited null association with OS, HR = 1.26 (0.96 - 1.66), *P*= 0.096 (Figure 5B); HR =2.43 (0.34 - 17.29), *P*=0.36 (Figure 5C).

**Figure 6:**
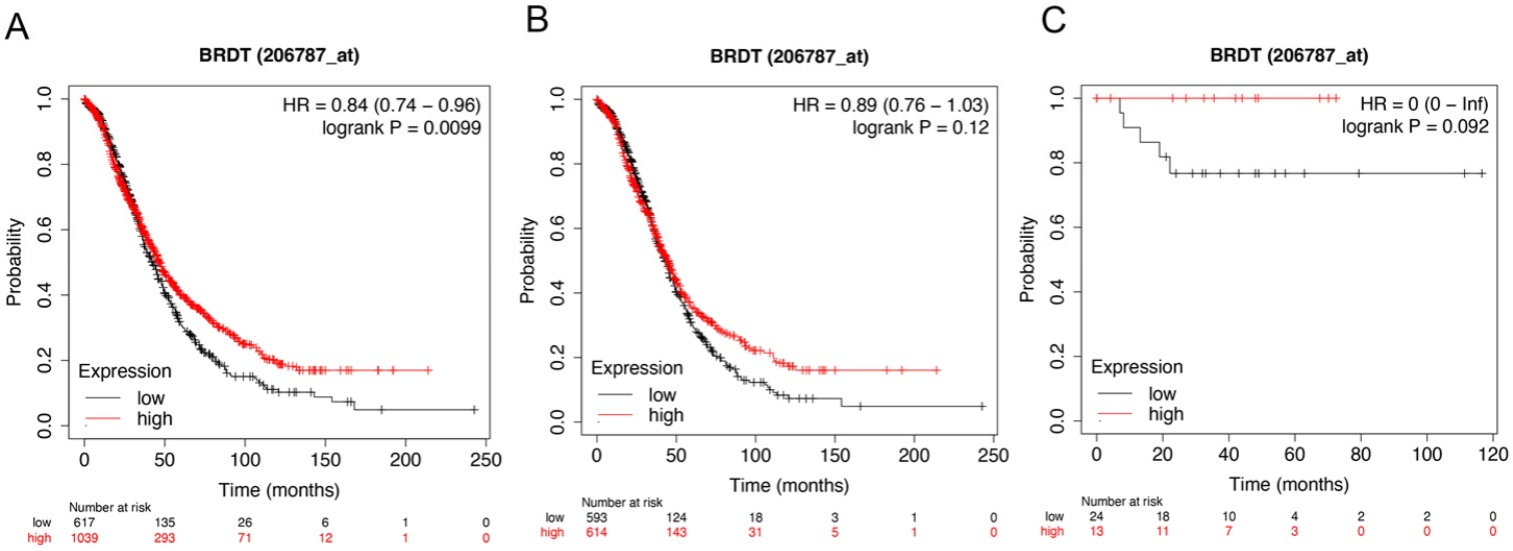
** The prognostic value of *BRDT* expression in ovarian cancer.** The prognostic value of *BRDT* expression in www.kmplot.com. Affymetrix ID for *BRDT*: 206787_at. OS curves were plotted for (**A**) all the patients (*n*= 1656), (**B**) serous cancer patients (*n*= 1207), and (**C**) endometrioid cancer patients (*n*= 37).

Figure 6 showed the prognostic significance of BRDT mRNA expression in the database. The desired Affymetrix ID: 206787_at. The over expression of BRDT mRNA was discovered to be considerably linked with better OS for all ovarian carcinoma patients, HR= 0.84 (0.74 - 0.96), *P* = 0.0099 (Figure 6A). However, mRNA expression of BRDT in serous and endometrioid cancer patients revealed no any association with OS in ovarian cancer, HR= 0.89 (0.76 - 1.03), *P* = 0.12 (Figure 6B); HR= 0 (0 - inf), *P* = 0.092 (Figure 6C), respectively.

Additionally, the correlation of BET protein sub-members with various clinicopathological types was further investigated, including pathological grades (Table 1), clinical stages (Table 2), TP53 mutation (Table 3), and chemotherapy types (Table 4) of ovarian cancer patients. As presented in Table 1, high expression of BRD2 mRNA in grade II and grade III was correlated to a better OS in patients with ovarian cancer. Furthermore, overexpression of BRDT mRNA was linked with a better OS in grade I and grade III, patients with ovarian carcinoma. However, high BRD2 mRNA expression in grade I and BRD4 mRNA in grade III showed significantly poor OS in patients with ovarian cancer. Moreover, high BRD3 mRNA expression indicated no correlation with OS in ovarian cancer patients.

Concerning clinical stages, BRD2 and BRD3 in advanced stage (III+IV), and BRDT in all clinical stages (I+II, III+IV) were associated with the better OS in ovarian cancer patients, although high BRD4 mRNA level showed worse OS in early-stage (I+II) patients with ovarian cancer (Table 2).

We additional studied the association amongst individual BETs and the prognosis of ovarian carcinoma according to the TP53 status (Table 3). Our results showed that BRD2 mRNA implied an improved OS in TP53 mutated ovarian malignancy patients. However, BRD3, BRD4, and BRDT mRNA expression showed null association in both TP53 mutated and wild ovarian malignancy patients.

In addition, subgroup survival analysis of ovarian cancer patients with various treatment strategies (Table 4) illustrated that overexpression of BRD2 mRNA was significantly correlated with an improved OS for patients treated with Platin-based chemotherapy compared with low expression. In addition, overexpression of BRD3 and BRDT mRNA was associated to a better-quality OS for patients with ovarian carcinoma receiving Taxol and concurrent Taxol+Platin-based chemotherapy. Whereas, high expression of BRD4 mRNA expression showed no correlation with any of the chemotherapy in ovarian cancer patients.

### HPA

**Figure 7:**
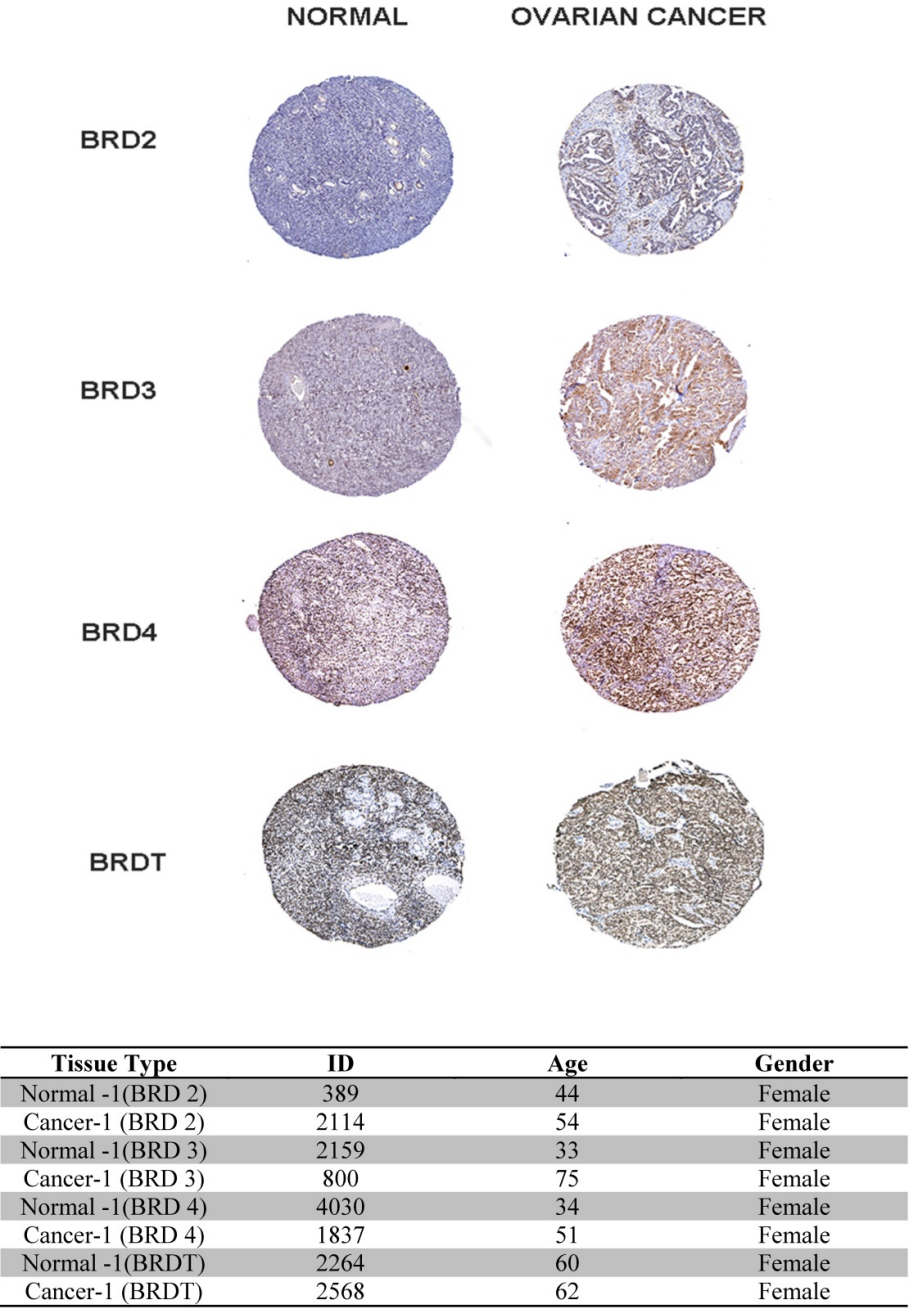
Immunohistochemistry image as obtained from HPA: The selected image of BET proteins detected in the HPA database showed trends toward differential expression in normal and breast cancer tissues in 400x magnification. The table showed the basic information of the IHC slices.

In our study, (Figure 7) the BET family genes protein expression in ovarian cancer tissues and normal ovary tissues are reviewed in the Human Protein Atlas. Summarizing the results of immunohistochemistry staining (IHC staining), for BET, we observe that among 4 cancer tissues examined. The database adopts two kinds of antibodies for the immunohistochemistry staining (CAB and HPA antibodies). In this investigation, we found that the BRD4 proteins were not vented in normal and ovarian cancer tissues. Despite this, BRD2, BRD3, and BRDT expression were discovered to be low in normal breast tissues, while it was shown to be high and medium in the cytoplasmic and membranous parts of breast tumor tissues, respectively. Finally, the database information for the BET IHC slides is listed in the table below.

### The various BRD2, BRD3, BRD4, and BRDT mRNA expression amongst ovarian cancer cells and normal ovarian cell

**Figure 8:**
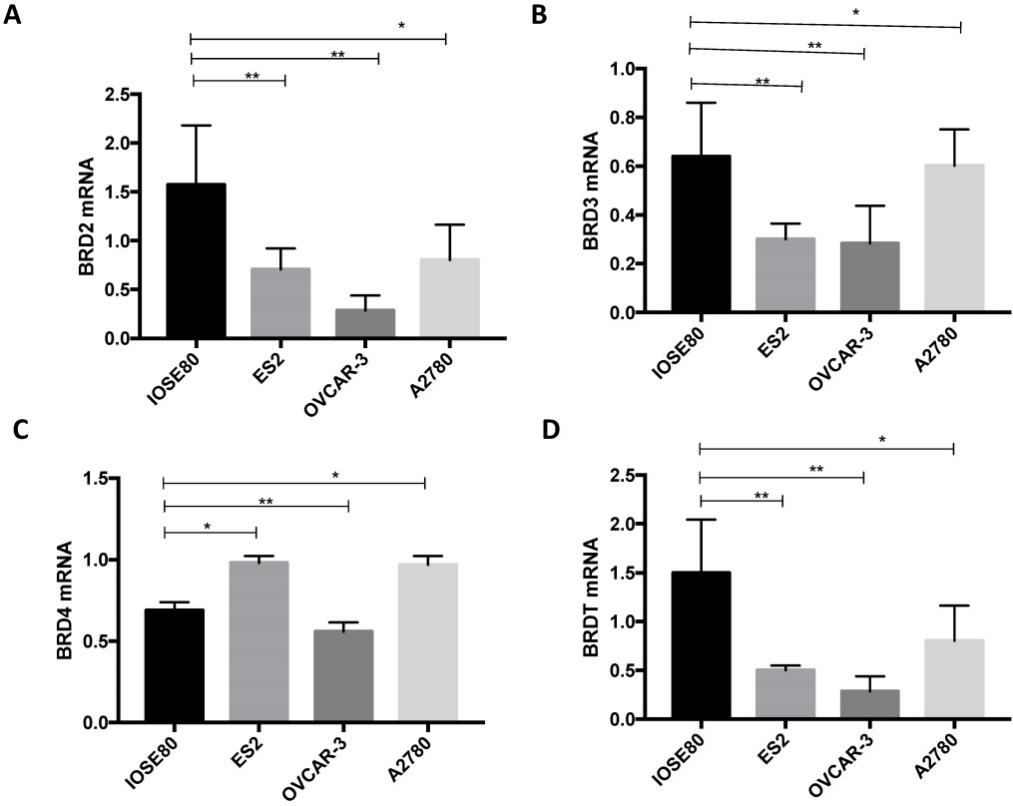
Analysis of (A) BRD2, (B) BRD3, (C) BRD4, (D) BRDT mRNA expression levels in breast cancer cells and normal breast cancer cells using qRT-PCR. **P<0.05.

As demonstrated in Figure 8, the BRD2, BRD3, and BRDT mRNA expression in the human ovarian cancer cell lines were all considerably downregulated in relationship with those in a normal ovarian cell line (P < 0.05). Furthermore, mRNA expression of BRD4 was notably lower in the normal ovarian cell than that in ovarian cancer ES2 and A2780 cell lines, but considerably greater than that in ovarian cancer OVCAR-3 cell line (P < 0.05).

### BET protein expression in breast cancer cells and normal ovarian cells

**Figure 9:**
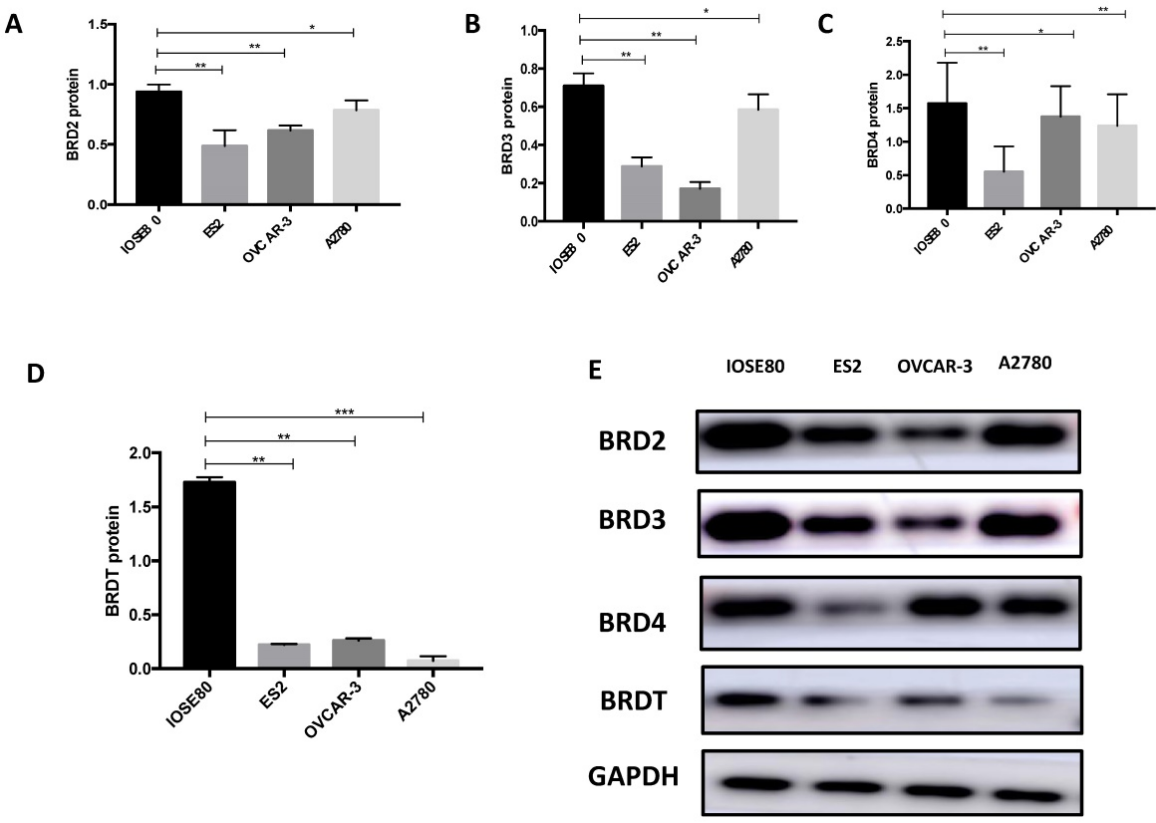
The protein expression of (A) BRD2, (B) BRD3, (C) BRD4 and (D) BRDT were detected in ovarian cancer cells ad normal cell using western blot, and (E) showed the western blot ban ds. **P<0.05, ***P<0.01.

Our results (Figure 9) showed that the protein level of BRD2, BRD3, BRD4, and BRDT in ovarian cancer cell lines was all subsided associated to the normal breast cell (*P* < 0.05).

## Discussion

According to our recent study, we broadly examined the prognostic roles of mRNA expression in ovarian malignancy patients of BET family by means of the KM plotter database. We observed that high levels of BRD2, BRD3, and BRDT indicated a better prognosis, whereas BRD4 predicted poor clinical outcomes in patients with ovarian carcinoma.

As a member of the BET family, BRD2 is expressed ubiquitously and binds to acetylated histone tails through the double bromodomain. BRD2 has been reported to play vital roles in the regulation of transcription and cell cycle progression 10, 14, 15. Moreover, previous studies have shown that BRD2 was essential for cell cycle exit and neuronal variation in mouse neuroepithelial cells, BRD2-deficient embryos presented embryonic lethality and cranial neural tube closure defects in the mouse 16-18. But so far, the expression and its prognostic role in ovarian carcinoma have not yet been discovered. In this study, we discovered that BRD2 mRNA expression showed better OS in all the ovarian malignancy patients, serous ovarian cancer patients, advanced clinical stage (III and IV), pathological grade (II and III) ovarian cancer patients as well as all patients treated with Platin based chemotherapy, suggesting that BRD2 represent a favorable prognostic marker for patients with ovarian malignancy, specifically in poor differentiation and advanced clinical stage serous ovarian cancer patients.

BET family protein BRD3, an acetylated GATA1 interacting partner, assists as a reader of acetyl symbols as an important hematopoietic transcription factor to endorse its chromatin tenancy at erythroid end genes 19. Nonetheless, its expression and role in ovarian cancer have been largely unknown. Current survival analysis outcomes indicated that mRNA expression BRD3 was related with longer survival in endometrioid ovarian carcinoma and advanced stage (III+IV) ovarian malignancy patients, together with patients with ovarian cancer, received Taxol and concurrent Taxol+Platin based chemotherapy, implying that this gene could be a favorable prognosis marker in ovarian cancer patients.

BRD4 is the mostly reviewed member of the BET family. Several reports have shown that BRD4 expression was significantly upregulated in different kinds of tumors, and BRD4 played vital role in the progression of cancers. Liao et al. 9 found that the BRD4 level was increased in non-small cell lung carcinoma, suppressing of BRD4 contributed to the reserve of cell proliferation and invasion, acceleration of cell apoptosis. In addition, they detected that BRD4 overexpression was associated to histological type and malignant behaviors (lymph node metastasis, tumor differentiation, and stage) of lung cancer. More importantly, they demonstrated that high BRD4 expression was associated with a poor prognosis for patients with lung cancer. Yan et al. 20 reported that the BRD4 mRNA expression and protein in urothelial malignancy of the bladder was higher when compared with normal bladder tissues; furthermore, BRD4 expression was correlated to histological grade, lymph node metastasis, and distant metastasis. They further conducted survival analysis and concluded that BRD4 expression predicted poor prognosis in bladder cancer patients. In patients with ovarian cancer, Ucar et al. discovered that somatic augmentation of BRD4 was associated with elevated mRNA levels BRD4 and suggestively linked with poor overall and progression-free survival associated to wild-type cases by using clinical and genomic records from The Cancer Genome Atlas 21. Consistently, according to the Kaplan Meier survival curves analysis, we also found that mRNA expression BRD4 was related with poor OS in all ovarian cancer patients, pathological grade III ovarian malignancy patients, and early clinical stage (I+II) ovarian malignancy patients. Based on prior indication in addition to our results, we can accept that BRD4 was a poor prognosis indicator in ovarian malignancy patients.

Unlike the other three BET proteins, BRDT expression is testis-specific in the normal adult human 22. However, a previous study has shown that BRDT was also stated in some cancer tissues. Using standard RT-PCR expression analysis, Scanlan et al. 23 observed that the BRDT gene was aberrantly activated in 12 of 47 cases of non-small cell lung cancers, 1 of 12 cases of squamous cell carcinoma of the head and neck, and 1 of 12 cases of esophageal squamous cell carcinoma. However, they did not notice any initiation of BRDT in colon cancer, melanoma, breast cancer, bladder, and kidney tumors. But so far, its expression and prognostic value in ovarian malignancy have not been researched. In the current study, we discovered that BRDT mRNA expression was linked with longer overall survival in all ovarian cancer patients, grade I and grade III ovarian cancer patients, all clinical stage (I+II, III+IV) ovarian cancer patients as well as all patients cured with Taxol and concurrent Taxol+Platin chemotherapy. Taken together, our outcomes indicated that BRDT expression was linked with improved prognosis in ovarian malignancy patients.

Furthermore, it has been identified that inhibition of BRD can promote full neoplastic transformation in the presence of wild-type p53. However, crosstalk between individual BET members and TP53 gene status in ovarian cancer has limited reports. The present data source has shown that BRD2 high mRNA expression has a promising OS in TP53 mutated ovarian cancer patients. Although, BRD3, BRD4, and BRDT high mRNA expression showed a negative correlation with TP53 mutated type of ovarian cancer. As the sample size of TP53 wild-type ovarian cancer patients was small, we could not establish the prognostic significance of the BET member in these subtypes' ovarian malignancy, further study including a large sample size may validate the findings.

Our study was the first to study the BET family's predictive effect in ovarian cancer patients. Conversely, there were several restrictions to our research that should be considered. First, we did not investigate the prognostic functions of four BET members (BRD2,3,4, and BRDT) in ovarian cancer. Second, the mechanism by which BRD2, 3, and BRDT were linked to a better survival consequence and stated differently in ovarian malignancy and normal ovarian cells/tissues was not found, which would be the subject of future analysis in our lab. Finally, our research focused on the distinct mRNA and protein expression of BET members in cell lines rather than tissue samples. In its place, we used the various expressions of BET members, which will be further investigated using RT-PCR and western blot to investigate BET expression in ovarian cancer patients.

## Conclusion

In summary, by performing the database targeted mRNA functional analyses, we have identified that high expression of BRD2, BRD3, and BRDT predicted a better prognosis, whereas BRD4 expression showed poor prognosis in ovarian malignancy patients. The results of the present study suggest that BET family mRNA expression is associated with ovarian cancer prognosis; furthermore, individual BET family genes could act as a predictive prognostic indicator in ovarian cancer.

## Figures and Tables

**Table 1:** Correlation of BET gene mRNA expression level with pathological grades of ovarian cancer patients

BET	Pathological grade	Cases	HR (95% Cl)	*P*-value
BRD2	I	56	3.98 (0.91 - 17.39)	0.048*
	II	324	0.68 (0.48 - 0.96)	0.026*
	III	1015	0.76 (0.64 - 0.89)	0.00097*
BRD3	I	56	0.44 (0.14 - 1.38)	0.15
	II	324	0.81 (0.57 - 1.16)	0.25
	III	1015	0.85 (0.72 - 1)	0.054
BRD4	I	41	0.44 (0.14 - 1.35)	0.14
	II	162	1.23 (0.78 - 1.93)	0.37
	III	392	1.54 (1.18 - 2.01)	0.0015*
BRDT	I	56	0.33 (0.12 - 0.89)	0.022*
	II	324	0.78 (0.57 - 1.08)	0.13
	III	1015	0.81 (0.68 - 0.96)	0.018*

*P<0.05

**Table 2:** Correlation of BET gene mRNA expression level with clinical stages of ovarian cancer patients

BET	Clinical stages	Cases	HR (95% Cl)	*P*-value
BRD2	I+II	135	1.78 (0.82 - 3.85)	0.14
	III+IV	1220	0.77 (0.66 - 0.89)	0.00054*
BRD3	I+II	135	2.84 (0.85 - 9.48)	0.075
	III+IV	1220	0.82 (0.7 - 0.96)	0.012*
BRD4	I+II	83	2.81 (0.97 - 8.12)	0.048*
	III+IV	487	1.23 (0.95 - 1.61)	0.12
BRDT	I+II	135	0.36 (0.16 - 0.81)	0.0097*
	III+IV	1220	0.81 (0.69 - 0.95)	0.0097*

*P*<0.05

**Table 3:** Correlation of BET gene mRNA expression level with TP53 mutation of ovarian cancer patients

BET	TP53 mutation	Cases	HR (95% Cl)	*P*-value
BRD2	Mutated type	506	0.6 (0.45 - 0.8)	0.00045*
	Wild type	94	0.62 (0.36 - 1.07)	0.081
BRD3	Mutated type	506	1.25 (0.95 - 1.64)	0.12
	Wild type	94	0.73 (0.42 - 1.26)	0.26
BRD4	Mutated type	124	1.3 (0.84 - 2.01)	0.24
	Wild type	19	Not available	Not available
BRDT	Mutated type	506	1.18 (0.91 - 1.54)	0.22
	Wild type	94	1.39 (0.8 - 2.41)	0.24

*P<0.05

**Table 4:** Correlation of BET gene mRNA expression level with applied chemotherapy for ovarian cancer patients

BET	Chemotherapy	Cases	HR (95% Cl)	P-value
BRD2	Contains Platin	1409	0.8 (0.69 - 0.92)	0.002*
	Contains Taxol	793	0.85 (0.69 - 1.06)	0.15
	Contains Platin +Taxol	776	1.22 (0.98 - 1.52)	0.069
BRD3	Contains Platin	1409	0.91 (0.79 - 1.04)	0.17
	Contains Taxol	793	0.82 (0.68 - 1)	0.044*
	Contains Platin +Taxol	776	0.82 (0.68 - 1)	0.045*
BRD4	Contains Platin	478	1.11 (0.85 - 1.45)	0.43
	Contains Taxol	357	1.25 (0.89 - 1.77)	0.2
	Contains Platin +Taxol	356	1.26 (0.89 - 1.78)	0.2
BRDT	Contains Platin	1409	0.87 (0.75 - 1.01)	0.061
	Contains Taxol	793	0.82 (0.68 - 0.99)	0.041*
	Contains Platin +Taxol	776	0.78 (0.64 - 0.95)	0.013*

*P<0.05

## Abbreviations

CI: confidence interval
CRD: carbohydrate-recognition domain
HR: hazard ratio
KM plotter: Kaplan-Meier plotter
BET: Bromodomain and extra-terminal
OS: overall survival
